# The Effectiveness of Injury Prevention Programs to Modify Risk Factors for Non-Contact Anterior Cruciate Ligament and Hamstring Injuries in Uninjured Team Sports Athletes: A Systematic Review

**DOI:** 10.1371/journal.pone.0155272

**Published:** 2016-05-12

**Authors:** Alireza Monajati, Eneko Larumbe-Zabala, Mark Goss-Sampson, Fernando Naclerio

**Affiliations:** 1 Department of Life and Sport Science, University of Greenwich. Medway Campus, Central Avenue, Chatham Maritime, Kent ME4 4TB, United Kingdom; 2 Clinical Research Institute, Texas Tech University Health Sciences Center, Lubbock, TX, United States of America; Sapienza University of Rome, ITALY

## Abstract

**Background:**

Hamstring strain and anterior cruciate ligament injuries are, respectively, the most prevalent and serious non-contact occurring injuries in team sports. Specific biomechanical and neuromuscular variables have been used to estimate the risk of incurring a non-contact injury in athletes.

**Objective:**

The aim of this study was to systematically review the evidences for the effectiveness of injury prevention protocols to modify biomechanical and neuromuscular anterior cruciate and/or hamstring injuries associated risk factors in uninjured team sport athletes.

**Data Sources:**

PubMed, Science Direct, Web of Science, Cochrane Libraries, U.S. National Institutes of Health clinicaltrials.gov, Sport Discuss and Google Scholar databases were searched for relevant journal articles published until March 2015. A manual review of relevant articles, authors, and journals, including bibliographies was performed from identified articles.

**Main Results:**

Nineteen studies were included in this review. Four assessment categories: i) landing, ii) side cutting, iii) stop-jump, and iv) muscle strength outcomes, were used to analyze the effectiveness of the preventive protocols. Eight studies using multifaceted interventions supported by video and/or technical feedback showed improvement in landing and/or stop-jump biomechanics, while no effects were observed on side-cutting maneuver. Additionally, multifaceted programs including hamstring eccentric exercises increased hamstring strength, hamstring to quadriceps functional ratio and/or promoted a shift of optimal knee flexion peak torque toward a more open angle position.

**Conclusions:**

Multifaceted programs, supported by proper video and/or technical feedback, including eccentric hamstring exercises would positively modify the biomechanical and or neuromuscular anterior cruciate and/or hamstring injury risk factors.

## Introduction

Hamstring strain (HAM) and anterior cruciate ligament (ACL) injuries are, respectively, the most prevalent [1] and serious [2] non-contact occurring injuries in team sports and therefore preventive programs aiming to protect athletes from both types of injury should be integrated. Several injury prevention programs involving jumps [3], strength [4–7], unstable [8,9], or a combination of different exercises modes [10–13] have been proposed to prevent both ACL and HAM injuries. However, there is still a lack of uniform criteria regarding the design of an ideal protocol for effective protection against the two aforementioned injuries in team sport athletes. Indeed, to the authors’ knowledge there is no consensus about how to integrate ACL and HAM preventive exercises within an optimal injury prevention protocol in team sports. A recently published systematic review highlights the lack of enough evidence to support the effect of neuromuscular training programs to reduce ACL injuries in athletes [2]. Additionally, it seems that multifaceted programs involving strength, plyometric, balance, agility, core, and flexibility exercises would be the most effective intervention to prevent from ACL injuries [2]. Similarly, effective strategies to reduce the incidence of HAM injuries may also include a combination of different types of muscular actions including both active lengthening eccentric and co-contracting knee stabilizer exercises [1,14].

In previously uninjured athletes the protective effects of different prevention protocols have been assessed by their capacity to modify biomechanical (posture, trunk, or lower limb alignments) and neuromuscular (strength deficits or balance) risk factors, rather than to reduce injury rates (the later require more time and also only can be accomplished through a prospective study). For example, knee valgus or varus moment and open knee flexion angle during landing, exaggerated hip internal rotation and adduction, and/or an uncontrolled trunk motion including lateral displacement during jumping [12,15], or cutting maneuvers [16] have been associated with an increased ACL injury risk in females athletes. On the other hand, the angle at which the optimal knee flexor peak torque occurred has been used to assess the risk of HAM injury [17]. Furthermore both ACL and HAM injuries have been associated with hamstring strength, hamstring-to-quadriceps strength ratio or hamstring bilateral ratio [18]. Even though the above-mentioned variables have been the focus of several trials [1,2,19], there is still a lack of consensus about how these factors would respond to different training interventions. For example, when strength training exercises were used alone, including closed-chain hip rotation, bands, machine and free weight lower body exercises, studies reported no change [5] to significant modifications [20] in the hip internal rotation, and knee abduction moment during running or cut and jump actions. Furthermore, significant increases in isometric hamstring strength in response to similar eccentric exercise protocols have been produced with [21] or without [22] a concomitant displacement of the optimal knee flexion peak torque toward a more open angle position.

To the authors’ knowledge there are still no standardized guidelines for designing an effective lower limb injury prevention protocol in terms of exercise modes (stable, balance, open or closed chain, using eccentric or concentric actions), sets, repetitions and relative overload in team sport athletes. Therefore, the aim of the current review is to examine the documented effects of the different proposed injury prevention protocols on the following modifiable ACL and/or HAM risk factors in uninjured team sport athletes: i) knee valgus/varus angle and moment; ii) hip adduction/abduction angle and moment; iii) knee and hip rotation angle; iv) knee and hip flexion angle; v) hamstring and quadriceps muscle strength; vi) hamstring to quadriceps (H/Q) conventional and functional strength ratios; and vii) the angle at which the optimal knee flexor peak torque occurred.

## Method

A systematic review of the literature was conducted in accordance with the PRISMA guidelines (S1 Table) [23,24] with procedures defined a priori. Search of literature was performed by using PubMed, Science Direct, Web of Science, Cochrane Libraries, U.S. National Institutes of Health clinicaltrials.gov, Sport Discuss and Google Scholar, from the start date of the representative database through the last week of March 2015. English-language publications in human populations were identified as being eligible for review. Articles were included if they were published in peer reviewed journals and full text was accessible. Commentaries, reviews, or duplicate publications from the same study were removed. Manual searches of personal files were conducted, along with screening of reference lists of previous reviews and identified articles, for inclusion. Combinations of the following keywords were used as search terms: “Anterior cruciate ligament or ACL and injury”; “hamstring and injury or strain”, together with the markers “exercise”, “intervention”, “training”, “protocol” “prevention” “muscle”, “biomechanics”, “kinetic”, and “kinematic”.

The selection criteria were applied independently by two reviewers (AM and FN). Potentially relevant articles were selected by: 1) screening the titles; 2) screening the abstracts; and 3) if abstracts did not provide sufficient data, the entire article was retrieved and screened to determine whether it met the inclusion criteria depicted in Table 1.

**Table 1:** Study Criteria for Inclusion in the Review.

Intervention studies
Duration of at least 4 weeks involving minimum of 8 training sessions no longer than 35 minutes
Examined at least one of the previously defined lower extremity injury risk factors
Involves male and/or female athletes (an athlete was defined as a person who performs minimum of two organized training sessions per week).
Participants: ≥14 years old, team sport athletes,
Without history of an ACL and/or hamstring injury, not engaged in any injury prevention program over the last 12 months prior to the intervention

The abstracts of the search results were reviewed. Reference lists of relevant studies were also reviewed to identify publications not found through the electronic search. Only studies examining the effect of injury prevention protocols on some of the previously identified HAM and/or ACL injury risk markers were considered. When data were not accurately presented (only available from figures or graphs) authors were contacted and requested to provide the appropriate range of values.

The following qualitative and quantitative information was extracted from each included study: authors; publication year; baseline population characteristics; intervention and control procedures; study duration; sample size per group; training modalities, number of exercises, sets, frequency and total time per session; outcomes measured at pre- and post-intervention; group means and SDs for the following variables: quadriceps and hamstrings strength; hip and knee flexion and extension moments; hip initial flexion and abduction angles; hip peak flexion and abduction angles; hip maximum external rotation angle; knee peak valgus moment; knee external rotation moment; knee Peak internal-rotation moment; knee initial flexion angle; knee peak flexion angle; knee valgus angle; optimal knee flexion peak torque localization; optimal knee extension peak torque localization and conventional and functional H/Q. In order to analyze the observed results using comparable assessment methods, the information was organized into four categories: i) landing, ii) side cutting, iii) stop-jump, and iv) muscle strength.

Methods of the analysis and inclusion criteria were specified in advance, and documented in a protocol registered at the International prospective register of systematic reviews, PROSPERO (CRD42015028041).

### Methodological assessment and risk of bias

Two reviewers (AM and FN) ascertained individual study information independently as part of the quality control process. The methodological quality of the included studies was assessed based on criteria adapted from Downs and Black [25]; Kennelly [26] and Physiotherapy Evidence Database (PEDro) scale: 1) clearly described the aim/hypothesis/objective; 2) participants free of previous knee/hamstring injury; 3) groups at baseline similar (sex, age and activity/sport); 4) clearly described characteristic of the participants; 5) clearly described Inclusion/exclusion criteria; 6) main outcome clearly described; 7) replicable (clearly described intervention protocol); 8) clearly presented results; 9) reported actual probability value for the main outcomes (e.g. 0.035 rather than <0.05); 10) staff, places and facilities where the participants were treated, representative of the treatment of the majority of the population; 11) availability of control group; 12) blinded researcher measuring the outcomes of the intervention; 13) patients from different intervention groups recruited over the same period of time; 14) randomized study; 15) incompliance reported; 16) reliability of outcomes. For each item, each study could be scored either 1 or 0 points. If the item was not applicable or not reported in the study, 0 points were recorded. For each study, the total quality assessment scored ranged from 0 to 16. Higher quality assessment number indicated a better methodological approach.

### Statistical analysis

From the collected data, we used the pre and post values of mean, standard deviation (SD), and sample size. The effect size was calculated using the Hedges’ g.

## Result

**Fig 1:**
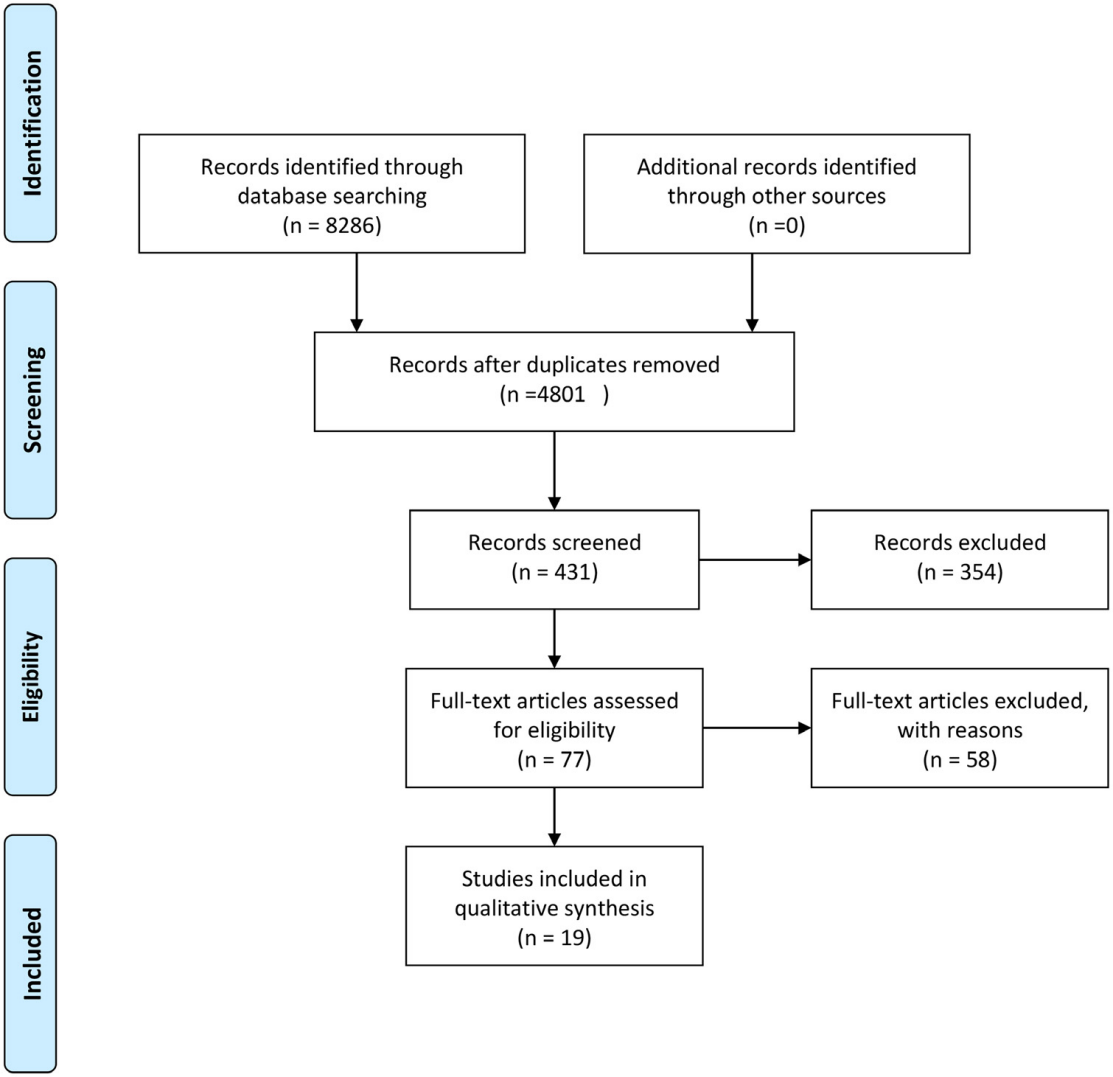
Flow diagram of article selection according to PRISMA.

After removing the duplicates, 4801 records were found through three electronic databases. Title and abstract selection excluded 4370 and 354 records, respectively. The remaining 77 records were reviewed based on exclusion/inclusion criteria and 56 studies were rejected for different reasons (Fig 1 and S2 Table). One of the reviewed studies was excluded because of using selective participants (high-risk vs. low-risk athletes) [27]. Another study was also excluded because of unclear intervention protocol [18]. Thus a total of 19 studies were included (Fig 1).

The scores for the methodological quality assessment ranged from 9 to 15 and the mean was 12.2 (Table 2).

**Table 2:** Quality assessment of the included studies.

Study	Quality score	1	2	3	4	5	6	7	8	9	10	11	12	13	14	15	16
Brughelli *et al*. [28] 2010	13	1	0	1	1	0	1	1	1	0	1	1	1	1	1	1	1
Chappel and Limpisvasti [29] 2008	9	1	0	0	1	1	1	1	1	1	1	0	0	0	NA	1	0
Clark *et al*. [30] 2005	10	1	1	1	0	1	1	1	1	1	1	0	0	1	NA	0	0
Daneshjoo *et al*. [31] 2012	13	1	1	1	1	0	1	1	1	0	1	1	1	1	1	0	1
Donnelly *et al*. [8] 2012	13	1	1	1	1	1	0	1	1	0	1	1	1	1	0	1	1
Herman *et al*. [6] 2008	14	1	1	1	1	1	1	1	1	0	1	1	0	1	1	1	1
Herrington [3] 2010	12	1	1	1	1	1	1	1	1	1	1	0	0	1	NA	0	1
Holcomb *et al*. [7] 2007	12	1	1	1	1	1	1	1	1	1	1	0	0	1	NA	0	1
Kato *et al*. [32] 2008	13	1	1	1	1	1	1	1	1	1	1	1	0	1	1	0	0
Lephart *et al*. [33] 2005	12	1	1	1	1	1	1	1	1	0	1	0	0	1	1	0	1
Lim *et al*. [11] 2009	13	1	1	1	1	1	1	1	1	0	1	1	0	1	1	0	1
Mendiguchia *et al*. [34] 2014	15	1	1	1	1	1	1	1	1	1	1	1	0	1	1	1	1
Mjølsnes *et al*. [35] 2004	14	1	1	1	0	1	1	1	1	1	1	0	1	1	1	1	1
Naclerio *et al*. [36] 2013	14	1	1	1	1	1	1	1	1	1	1	1	0	1	1	0	1
Nagano *et al*. [37] 2011	11	1	1	1	1	1	1	1	1	0	1	0	0	1	NA	0	1
Ortiz *et al*. [38] 2010	10	1	0	1	0	0	1	1	0	0	1	0	1	1	1	1	1
Pollard *et al*. [39] 2006	12	1	1	1	1	1	1	1	1	1	1	0	0	1	NA	1	0
Wilderman *et al*. [40] 2009	13	1	1	1	1	1	1	1	1	1	1	1	0	1	1	0	0
Zebis *et al*. [41] 2008	9	1	0	1	1	0	1	0	1	1	1	0	0	1	NA	0	1

Note: NA: not applicable; Quality score criteria are explained in the methodological assessment and risk of bias section.

The total number of participants in all included studies was 485, comprising 285 female and 200 male. The included articles used different protocols involving resistance [6], eccentric [30,35], or plyometric exercises [3] alone or combined with other exercise modalities [7,28,29,31,33,34,36,38–40] supported by video feedback [32] and/or technical corrections [8,11,37,41].

Two studies analyzed the effects of the applied interventions to modify some of the aforementioned risk factors during landing and stop-jump [3,29]; three studies considered landing and muscle strength outcomes [11,33,38]; one study evaluated stop-jump and muscle strength outcomes [6]; the rest of studies focused on a single test-task: landing [37,39]; stop-jump [32]; side cutting [8,40,41]; and muscle strength outcomes. [7,28,30,31,34–36]

Table 3 summarizes the type of intervention, main characteristics, and effects of the all-19 included studies.

**Table 3:** Summary of the main characteristics and relevant finding of the 19included studies.

Study	Assessment	Participants	Design and type of intervention	Length	Relevant findings
Chappel and Limpisvasti [29] 2008	Landing (DJ) and stop jump	Female (n = 30; 19±1.2 y) basketball (n = 18) and soccer (n = 12) players	Controlled within participants pre-post comparison. Ten exercises involving core, strengthening, dynamic joint stability and balance training, jump training, and plyometric exercises. With proper technical feedback, daily 10 to 15 minute workout.	6 wk	From DJ: ↓HIAbdA (g = -0.44); ↑KIFA (g = 0.54); ↑KPFA (g = 0.54); ↓KFM (g = -0.46) From stop jump: ↓HIFA (g = 0.68); ↓HMxERA (g = -0.52); ↓KERM (g = -0.26); ↓KPVM (g = -0.38) ↓KFM (g = -0.21)
Herrington [3] 2010	Landing (DJ) and stop jump	Female basketball players (n = 15; 19.1±6.1 y)	Controlled within participants pre-post comparison. Progressive jump training from bilateral to unilateral activities with proper feedback and technical corrections, 3-day per week 15 min session.	4 wk	↓ KVA at both limbs: DJ (left g = 1.54; right g = 1.74) and Stop Jump (left g = 0.73; right g = 0.54)
Lephart *et al*. [33] 2005	Landing (VJ) and muscle strength (isokinetic)	Female basketball or soccer players (n = 27; 14.3±1.3 y)	Two PG, randomized pre-post comparison. Weeks 1^st^ to 4^th^: Resistance flexibility and balance exercises for both groups. Weeks 5^th^ to 8, different interventions 1) Plyometric + agility (P, n = 14) 2) Basic resistance + flexibility + balance exercises (B, n = 13), 3-day per week 30 min session programme supported with verbal and video feedback.	8 wk	Both groups (P and B): ↑QS at 60°/s-1 and 180°/s-1 ↑HIFA (P g = 1.08; B g = 0.24) ↑KPFA (P g = 0.92; B g = 0.42); ↓HFM (P g = -0.26; B; g = 0.17) ↓KFM (P g = 0.61; B g = -0.69) P group only: ↑HPFA (g = 0.77)
Lim *et al*. [11] 2009	Landing (RVJ) and muscle strength (isokinetic)	Female basketball players (n = 22; 15 to 17 y)	Two PG, randomized pre-post comparison. 1) Experimental (E, n = 11) Modified version of Mandelbaum’s Prevent Injury and Enhance Performance (PEP) Programme involving stretching, strengthening, plyometric and agility exercises supported by technical corrections. Daily 20 min session. 2) Control (C, n = 11) only regular training	8 wk	E group to pre and to C: ↑KPFA (g = 0.41; ↑KFM (g = 0.41); ↓KPEM (g = -0.95); ↓KVM (g = -0.69) ↓QS and ↑H %EMG (g = 0.84)
Ortiz *et al*. [38] 2010	Landing (SLDJ) and muscle strength (isometric)	Female soccer players (n = 30, 14 to 15 y)	Two PG, randomized pre-post comparison 1) Experimental (E, n = 14): Flexibility, strengthening and plyometric exercises 2) Control (C, n = 14) continue its regular practice and games. Two days/week, 20 to 25 min workout.	6 wk	From SLDJ: ↑KPEM; ↑ KPVM; NS = between groups ** ↑QS E group to pre and to C
Nagano *et al*. [37] 2011	Landing (SLDJ)	Female basketball players (n = 8, 19.4±0.7 y)	Controlled within participants pre-post comparison Plyometric, balance exercises and specific basketball skills (first 3-weeks focused to improve landing technique). Three days/week, 20 min workout.	5 wk	↑ KIFA (g = 2.21)
Pollard *et al*. [39] 2006	Landing (DJ)	Female soccer players (n = 18, 14 and 17 y)	Controlled within participants pre-post comparison. Prevent injury and enhance performance protocol involving flexibility, strengthening, plyometric and agility exercises supported by video feedback. Three days/week, 20 min session.	16 wk	↓HIRA (g = -0.71); ↑HPAbdA (g = -0.64)
Donnelly *et al*. [8] 2012	Side-cutting (planned and unplanned)	Males Australian football players (n = 34, >19 y)	Two PG, pre-post comparison. 1) Experimental (E, n = 14) balance, plyometric, agility exercises supported by feedback and technical corrections. 2) Contrast shadow training (ST, n = 20). Both groups trained 2 days/week, 20 min session first 18 weeks and 1 day/week from 17^th^ to 28^th^ week.	28 wk*	Both E and ST: ↓KPIRM for planed side cutting (g = -0.57); ↑KPVM for unplanned side cutting (g = 0.44).
Wilderman *et al*. [40] 2009	Side-cutting	Female basketball players (n = 30, 21.1±2.8 y)	Two PG, randomized pre-post comparison 1) Experimental (E, n = 15), progressive agility training program. Four days/week, 15 min session 2) Control (C, n = 15) no specialized agility training.	6 wk	Both E and C. No change in knee kinematic; ↑MH (g = 0.94); ↓VM (g = -0.49) activation during ground contact phase
Zebis *et al*. [41] 2008	Side-cutting	Female (n = 20, 26±3 y) handball (n = 8) and soccer (n = 12) players.	Controlled within participants pre-post comparison. Neuromuscular training with technical support to improve awareness and neuromuscular control during landing, cutting and jumping with simultaneous ball handling. Two days/week, 20 min workout	12 months	NS in knee and hip kinematic ↑ST and NS in Q activation
Herman *et al*. [6] 2008	Stop Jump and muscle strength (isometric)	Female recreational team sport athletes (n = 66, 18 to 30 y)	Two PG, randomized pre-post comparison. 1) Experimental (E, n = 33), strengthening exercise using resistance bands and balls. Three days/week, 45 min session. 2) Control (c, n = 33) no strength training.	9 wk	E group to pre and to C
Kato *et al*. [32] 2008	Stop Jump	Female basketball players (n = 20; 20.4±1.0 y)	Two PG, randomized pre-post comparison 1) Experimental (E, n = 10) Strengthening, jump-landing and balance exercises supported by feedback and technical corrections. Three days/week, 20 min session. 2) Control (C, n = 10) no intervention.	4 wk	E group to pre and to C ↓KVA (g = -1.50)
Naclerio *et al*. [36] 2013	Muscle strength (isometric)	Male professional soccer players (n = 20, 23.8±3.1 y)	Two PG randomize pre-post comparison. 1) E experimental (E, n = 10), strengthening eccentric and balance exercises. Performed 3 day/week 15 min session 2) control (C, n = 10) no intervention.	4 wk	E group to pre and to C; ↑H isometric PT at 800 (g = 0.78) and 35°(g = 0.50) knee angles
Brughelli *et al*. [28] 2010	Muscle strength (isometric)	Male football players (n = 28, 21.1±1.4)	Two PG randomized pre-post comparison. 1) Experimental (E, n = 13) Strengthening eccentric exercise program. Three days/week, 15min session. 2) Control (C, n = 11) only regular football training.	4 wk	Both groups: ↑ KFPTL (E g = 1.10 C g = 0.74) E:↑ OKEPTL (g = 0.87)
Clark *et al*. [30] 2005	Muscle strength (isokinetic)	Male Australian Rules football players (n = 9, >18 y)	Controlled within participants pre-post comparison. Progressive eccentric training involving only the Nordic Curl exercise (2 to 3 sets of 5 to 8 repetitions), 2–3 days/week	4 wk	↓QS at 60°/s^-1^ (dominant g = -1.1; non-dominant g = -1); ↑OKFPTL (dominant g = 0.63; non-dominant g = 0.95)
Holcomb *et al*. [7] 2007	Muscle strength (isokinetic)	Female soccer players (n = 12; 20± 0.8 y)	Controlled within participants pre-post comparison. Upper-body resistance exercises combined with speed and agility (2 days) and lower body (hamstring emphasized) resistance exercises combined with endurance conditioning training (2 days). Four days/week.	6 wk	↑H/Q functional ratio (average from concentric 240, 180, and 60°/s^-1^ and eccentric 60, 180, and 240°/s^-1^; g = 1.19)
Daneshjoo *et al*. [31] 2012	Muscle strength (isokinetic)	Male, soccer players (n = 36, 17 to 20 y)	Three PG randomized pre-post comparison. 1) FIFA+11 (F, n = 12), involving strengthening, balance, plyometric and agility exercises 2) Harmoknee (H, n = 12) involving strengthening and balance exercises 3) control (C, n = 12) regular training and warm up. Both F and H consisted in 3 days/week (24 sessions), 20 to 25min workout.	8 wk	F: ↑H/Q conventional ratio (g = 0.99); and ↓H/Q (g = -1.17) functional ratio, from pre to post NS in H and C
Mendiguchia *et al*. [34] 2014	Muscle strength (isokinetic)	Males soccer players (n = 51)	Two PG randomized pre-post comparison **1)** Experimental (E, n = 27) Neuromuscular protocol involving eccentric hamstring muscle strength, plyometric, and accelerations 2) Control (C, n = 24) only football. Intervention consisted in 2 days/week (14 sessions), 30 to 35 min workout before the soccer session.	7 wk	↑HS (E, Con D g = 0.71, Non-D g = 0.69; ECC D g = 0.98, Non-D g = 0.70) ↑H/Q conventional ratio; (E, D g = 0.62, Non-D g = 0.60) and functional ratio (E, D g = 0.99, Non-D g = 0.48)
Mjølsnes *et al*. [35] 2004	Muscle strength (isometric and isokinetic)	Male soccer players (n = 22, >18 y)	Two PG randomized pre-post comparison. 1) Nordic eccentric hamstring (NEH, n = 11), 2) Concentric hamstring (CH, n = 10). Progressive training from 2 sets of 6 reps to 3 sets of 8 to 12 reps over 4 weeks, and then increasing load for the final 6 weeks	10 wk	NEH: ↑HS eccentric at 60°/s^-1^ (g = 2.16) ↑isometric at 30° (g = 1.86) 60° (g = 1.32) and 90° (g = 1.84) ↑H/Q functional ratio (g = 1.99) NS in CH

Notes: ↑ increase; ↓ decrease; PG: parallel groups; NS: no significant differences, Sig = significant differences. %EMG = percentage of electromyography activity; H = hamstring, MH = medial hamstring; Q = quadriceps; VM = vastus medialis; ST = semitendinosus; H/Q = hamstring to quadriceps ratio; QS = quadriceps strength, HS = hamstrings strength; PT = peak torque; DJ = Drop Jump; SLDJ = single legged drop jump; RVJ = Rebound vertical jump; VJ = Vertical Jump; HIFA = hip initial flexion angle; HPFA = hip peak flexion angle; HIAbdA = hip initial abduction angle; HPAbdA = hip peak abduction angle; HMxERA = hip maximum external rotation angle; HIerRA; HFM = hip flexion moment. KIFA = knee initial flexion angle; KPFA knee peak flexion angle; KVA; knee valgus angle KFM = knee flexion moment; KERM = knee external rotation moment; KPIRM = knee Peak internal-rotation moment; KPEM = knee peak extension moment; KPVM = knee peak valgus moment; OKFPTL = optimal knee flexion peak torque localisation OKEPTL = optimal knee extension peak torque localization.

* test 1 was performed between weeks 1 (pre) to 7 and test 2 (post) between week 18 to 25 during the 28-week intervention period.

** Missing information impeded the calculation of g values

### Landing

Seven studies including only female participants, n = 143 (77 basketball and 66 soccer players) used plyometric combined with other exercise modalities (balance, strengthening and flexibility) to analyze the effects of injury prevention programs on kinematic and kinetic variables during landing [3,11,29,33,37–39]. Three studies analyzed a 30 cm drop vertical jump (DVJ) [3,29,39], two a vertical jump (VJ) [11,33], and the other two a 30 to 33 cm singled leg drop jump (SLD) [37,38]. The averaged quality of these studies was 11.5, ranging from 9 to 14, with 1 study scoring 14 (out of 16). Interventions lasted from 5 to 16 weeks.

Knee flexion angle increased after performing mixed interventions combining strength-balance and plyometric exercises [29,33,37] or following a program aiming to improve technique [11]. Conversely, no significant changes on knee flexion angle have been reported after performing both a 6-week [38] or a 16-week [39] mixed protocol in female soccer players.

Knee flexion moment was decreased in two studies where the intervention protocols involved active feedback aiming to improve the correct execution of selected balance exercises [29,33]. Only one study involving a 4-week progressive jump training reported significantly decreased and large effect sizes in valgus angle during landing [3], while no changes were observed by other 4 studies in which multifaceted interventions including plyometric, strengthening and balance exercises were implemented [29,37–39].

### Side-Cutting

Three studies involving 84 athletes (34 male and 50 female) analyzed the effectiveness of different injury prevention protocols to modify knee biomechanics during side-cutting maneuvers [8,40,41]. The mean quality score was 11.5, ranging from 9 to 13 (out of 16). Interventions lasted from 6 weeks to 12 months.

Two studies investigated 45° pivoting [8,40] and the other study did not report the pivoting angle [41]. All three studies focused on knee flexion angles and moments. The prevention programs varied between studies from a progressive agility exercise protocol [40] toward a combination based on feedback protocols including balance, plyometric and agility exercise, [8] and a proprioceptive-balance program [41]. The applied interventions did not increase knee flexion angles and moments measured during cutting maneuver. Two studies examined the effect on vertical ground reaction forces, but again interventions did not alter this variable when performing either pre-planned[8,40] and unplanned sidestepping actions [8].

### Stop-jump

Four studies involving a total of 131 female athletes, investigated the effect of exercise programs on kinematic and kinetic variables during double leg stop-jump (DLSJ) [3,5,29,32]. The average quality score was 12, ranged from 9 to 14 (out of 16). The interventions lasted 4 to 9 weeks.

Two studies performed the DLSJ after basketball drills [3,32]. Participants dribbled a basketball to free throw line and then performed a jump shot. For the other two studies participants take a three or four steps approach to run as fast as they felt comfortable followed by two-footed landing and a maximum height two-footed takeoff [6,29].

Knee valgus angle was reduced as a result of a four-week progressive jump training program [3] or a mixed intervention involving strength and balance exercises assisted by a video feedback protocol [32]. Furthermore, Chappell and Limpisvasti [29] reported significant reduction of both knee valgus moment and hip flexion angle as consequence of a 6-week strength, balance, plyometric and agility program involving a constant monitoring of the proper technique execution. Only one of the aforementioned four studies did not report any significant modification in knee and hip biomechanics during a stop-jump after a 9-week strength training intervention using bands and balls in female athletes [6].

### Muscle strength

Eleven trials involving 316 athletes (150 female and 166 male) reported the effects of exercise interventions on lower limb strength. Three studies considered only maximal isometric peak torques [6,36,38], seven studies measured isokinetic strength [7,11,28,30,31,33,34] and only one study measured both isometric peak torques and isokinetic force [35]. In addition, four of the aforementioned studies analyzed the effect of intervention on H/Q [28,30,31,34,35] and only two monitored changes on the optimal knee flexor peak torque localization [28,30]. The average quality score was 12.7, ranging from 10 to 15 (out of 16). The interventions lasted 4 to 10 weeks.

Both conventional and functional H/Q ratios increased after a 7-weeek neuromuscular multifaceted (plyometric, eccentric and acceleration exercises) program [34]. Additionally, functional H/Q ratio was also increased after a 4-week Nordic eccentric hamstring protocol in male soccer players [35], and also following a 6-week strength program including at least two different hamstring concentric exercises in females soccer players [7]. However, the latest study did not result in significant modification of the conventional H/Q ratio. One study involving only male athletes examined the FIFA11+ and the HarmoKnee protocols. The FIFA11+ increased the conventional H/Q ratio only in the dominant leg but both protocols decreased the functional H/Q ratio [31]. Furthermore, no changes in the conventional H/Q ratio were observed after performing a 4-week eccentric exercise protocol involving different open or closed kinetic chain and antagonistic exercises [28]. Two studies reported a shift to the optimal knee flexor peak torque toward to a more open angle position following a 4-week eccentric exercise intervention [28,30].

## Discussion

The main finding of the current review is that multifaceted programs including plyometric, balance, strength and/or agility exercises supported by appropriate feedback and technical indications seem to be more effective to positively modify biomechanical risk factors than protocols with no technical feedback, or involving only one mode of exercise. Furthermore, interventions using mainly strengthening exercises would improve muscle strength, H/Q ratios and/or promote a shift of optimal knee flexion peak torque toward a more open angle position, without further biomechanical modifications.

### Landing

Kinetics and kinematics of the lower extremity during landing from vertical or rebound jumps, and from drop jump seem to be more modifiable compared to other testing maneuvers such as side-cutting or stop-jump. Multifaceted interventions involving strengthening, balance, flexibility, plyometric or agility exercises, supported by appropriate feedback and technical corrections showed to be effective to improve hip [29,33,39] and knee [3,11,29,33] biomechanics (Table 3). Conversely, when no feedback was used, less clear effects on knee kinetics during landing from single leg drop jump were observed [38]. Indeed, a non-desirable increase of knee initial flexion angle during landing from single legged drop jump was observed after performing a protocol including plyometric and balance exercises with no technical feedback [37]. The lack of feedback and/or proper technical support during an unstable 1-leg landing task could have been the reason of the observed results. Furthermore, the improvements on landing technique after performing a 4-week protocol involving resistance, flexibility and balance exercises supported by verbal and video feedback did not ameliorate when a subsequent 4-week plyometric and agility protocol was implemented [33]. Nonetheless, Herrington [3], observed a significant decrease of the knee valgus angle during landing from drop and stop-jump in female athletes after performing a 4-week progressive jump training program supported with proper verbal and technique feedback.

Results from the previous investigations support the importance of proper feedback and technical correction to successfully improve landing biomechanics when performing protocols including different exercise modalities.

### Side-cutting

All of the included studies reported no effects of the injury prevention protocols to modify lower limb biomechanics during side-cutting maneuvers. Donnelly *et al*. [8] used a two parallel group design to compare the effectiveness of an intervention including balance, plyometric, agility exercises supported by feedback and technical corrections to a contrast shadow-training group. Although positive changes on the knee biomechanics during planned and unplanned side cutting maneuvers were observed, both protocols were equally effective, and therefore no advantage of implementing the preventive intervention was determined. Possibly, the low supervisor-participants ratio (1:40) together with the lack of specific side-cutting exercises including in the preventive protocol would explain the achieved results. Additionally, Wilderman *et al*. [40] reported no effect of a 6-week progressive agility training to modify knee kinematics during a 45° side-step pivot maneuver. Perhaps the absence of specific exercises to address knee and hip flexion angles and the lack of feedback in regard to the knee and hip alignments would be the cause of the unsuccessful results. Moreover Zebis *et al*. [41] were also unable to observe positive modification on a side-cutting maneuver after performing an 18-week neuromuscular protocol in elite handball and soccer female players. Maybe the high level of performance of the participants would have impeded further biomechanical improvements on the selected side cutting exercises.

In summary, an effective protocol to improve lower limb biomechanics during side cutting maneuvers remains to be elucidated.

### Stop-jump

Three studies using a 4-week [3,32] or a 6-week [29] multifaceted protocol including jumps and plyometric exercises combined with proper technical feedback improved knee valgus angle [3,32] and moment [29] during stop-jump. Conversely, a 9-week resistance-training program with no technical feedback, although effective to increase quadriceps and hamstring strength, did not produce any biomechanical modification during stop-jump [5]. The ineffectiveness of strength training alone to improve lower limb biomechanics during jump-related exercises was also observed in other studies [42,43]. Nevertheless, meaningful biomechanical improvements have been observed when strength protocols are combined with proper technical instructions and feedback [5].

The above-mentioned studies support the notion of combining sport-specific exercises with proper technical feedback to promote correct execution and biomechanical improvements during stop-jump. In addition, the positive effect of strength training maybe amplified by proper technical support to the sports-specific actions.

### Muscle strength

Eleven studies investigated the effect of resistance exercises alone [6,28,30], combined with balance [36], agility, speed [7], flexibility, jump [33,38], plyometric and sprint training [34] or integrated within an standardized injury prevention protocol such as FIFA11+, Harmoknee[31] or Mandelbaum’s Prevent Injury and Enhance Performance [11], Two interventions [30,35] using only the eccentric Nordic curl, improved hamstring strength along with a shift of the knee flexors maximal peak torque toward a more open angle position [30] and increase the functional H:Q ratio [35]. Further increases on the hamstring torque relationship were reported when this particular exercise was combined with an eccentric (single-leg dead lifts) and an unstable closed chain exercise (forward lunges on a Bosu^®^ balance trainer).[36] Additionally, substantial improvements in the functional H/Q ratio were observed after a 7-week neuromuscular protocol involving two eccentric exercises (Nordic hamstring and dead lift), plyometric and sprints.[34] This multifaceted intervention induced twofold to threefold lower increases in quadriceps peak torque than in hamstring peak torque and consequently eliciting a meaningful increase of the functional H/Q ratio from 0.89 to 1.0.

A shift in maximal peak torque occurring at a more open knee angle position during both isokinetic flexion (+4°) and extension (+6.5°) was also observed as a results of a 4-week strengthening program where the Nordic curl was combined with three predominantly quadriceps eccentric closed kinetic chain exercises.[28] Conversely, Holcomb *et al*. [7] reported meaningful increases of the H/Q ratios, especially at greater velocities, in a group of female soccer players after performing a 6-week of a multifaceted program including concentric but no eccentric hamstring exercises. As females have weaker hamstrings than men [44], it could be possible that in this particular group of female soccer players, no regular resistance training exercisers, a strengthening protocol with no particular eccentric hamstring components would be enough to initially improve hamstring activation and diminish disproportionate quadriceps force imbalance. Indeed similar results were observed by Herman [6] in female team sport athletes, with no regular resistance training, who increased hamstring and quadriceps isometric strength after a 9-week resistance bands and exercise balls protocol including no hamstring eccentric exercises.

Only Daneshjoo *et al*. [31] reported a non-desirable decrease of the H:Q functional ratio in both dominant and non-dominant limbs in male soccer players. This study analyzed the impact of two specific injury prevention programs (Harmoknee and FIFA11+) on conventional and functional H:Q ratio. Although no significant alterations were observed in the control and Harmoknee groups, participants allocated to the FIFA11+ showed a significant drop of the functional H:Q ratio from 0.83 to 0.49. The latest figures fall well below the recommended minimum threshold values of 0.89 on Biodex isokinetic dynamometer for preventing ACL injury in athletes [7]. Although both Harmoknee and FIFA11+ protocols include different types of strengthening, balance, running, plyometric and agility exercises, FIFA11+ involves greater knee extension components along with a relative lower emphasis on hamstring eccentric movements (only 1 set of 3 to 15 repetitions of Nordic curl) and therefore would be emphasizing quadriceps concentric over hamstring eccentric actions. Additionally, the interventions used in this particular study have taken place during the competition period with no preseason component. This sequence has shown to be detrimental to attenuate the incidence of ACL injury in female athletes [2]. Similarly Lephart *et al*. [33] reported a selective increase of quadriceps but not hamstring maximal peak torque in female team sport athletes after performing a multifaceted intervention excluding hamstring eccentric exercises. Conversely, Lim *et al*. [11] using another mixed protocol involving flexibility, plyometric, agility and strength exercises including 3 sets of 10 repetitions of Nordic curl, reported a reduction of quadriceps peak torque along with a positive increase of the hamstring activation during jumping in female basketball players. Although the influence of H/Q ratio as a risk factor for HAM injury has been questioned [45] lower values of both conventional and functional H/Q are still considered relevant risk factors for ACL injury [15]. Additionally, given the multifaceted etiology of both injuries the influence of H/Q ratios for increasing the risk of HAM and ACL injuries should not be ignored.

In summary, hamstring eccentric exercises such of Nordic curl, alone or integrated with other exercise modalities (unbalance, strengthening, plyometric, agility, sprint or flexibility) would improve hamstring strength and increase H/Q functional ratio along with or a shift of optimal knee flexion peak torque toward a more open angle position. Nevertheless, less strength-conditioned athletes would initially benefit from using multifaceted protocols including concentric hamstring, balance and other resistance exercises. Furthermore, in team sport involving a predominance of knee extension actions such as soccer or basketball it would be recommended to add hamstring eccentric exercises in order to balance the predominance of knee extension component resulted from the specific sport activities (e.g. jump-landing, stop-jump or side cutting maneuvers).

### Limitations and future studies

Seven studies were non-randomized single trials interventions [3,7,29,30,37,39,41], while one study [8] used a two parallel group non-randomized comparison. The lack of a parallel control group and randomization creates potential discordance among groups and introduces inherent selection bias that is difficult to ignore.

All the included studies focused on very specific and relatively homogeneous populations, e.g. male Australian Rules football players [30] male professional [28] or amateur [36] soccer players; female national league division I basketball players [3], etc. Maybe the specific training methods, including volume and intensity of different conditioning training, sport drills and competitive actions, body type, genetic variability, and other confounders would make it difficult to generalize results worldwide.

The uncertain effects of the analyzed risk factors to attenuate the incidence of both HAM and ACL injuries impede to make real assertions about the benefits of the used protocols to reduce the injury rate, rather than to elicit supposed beneficial alterations in some of the analyzed biomechanical and neuromuscular variables. In addition, from the analyzed studies, it was not possible to evaluate the duration of the effects and what would be the effective training dosage to maintain the obtained benefit over the complete season and between seasons. Futures studies using longer intervention periods lasting from more than 1 season should be designed in order to clarify proper dosage for maintaining and/or recover benefits on the analyzed modifiable injury risk factors in team sports athletes.

## Conclusions

Multifaceted programs including eccentric hamstring exercises combined with other training modalities such as plyometric, balance, resistance, agility and/or flexibility exercises would promote positive modifications on the previously identified HAM and ACL risk factors. The addition of appropriate technical feedback appears to be an essential component of the injury prevention protocols in team sport athletes.

## Supporting Information

S1 Table PRISMA Checklist. (DOC)

S2 Table Supporting information including the 56 excluded studies and reasons for exclusion. (DOCX)
