# Supplementary material for: The Effectiveness of Injury Prevention Programs to Modify Risk Factors for Non-Contact Anterior Cruciate Ligament and Hamstring Injuries in Uninjured Team Sports Athletes: A Systematic Review
Source: PLoS One. 2016 May 12;11(5):e0155272. doi: 10.1371/journal.pone.0155272 (PMC4865209; doi:10.1371/journal.pone.0155272)
Supplement: S2 Table — (DOCX) [file pone.0155272.s002.docx]

**Table S1. Summary of the 56 rejected studies and the corresponding reason for rejection**

|  | **Study** | **Reason for exclusion** |
| --- | --- | --- |
| 1 | Paterno MV, Myer GD, Ford KR, Hewett TE. Neuromuscular training improves single-limb stability in young female athletes. Journal of Orthopaedic & Sports Physical Therapy. 2004 Jun;34(6):305-16. | Intervention shorter than 4 weeks or sessions longer that 35 minutes |
| 2 | DiStefano LJ, Padua DA, Blackburn JT, Garrett WE, Guskiewicz KM, Marshall SW. Integrated injury prevention program improves balance and vertical jump height in children. The Journal of Strength & Conditioning Research. 2010 Feb 1;24(2):332-42. | Age of participant less than 14 years old |
| 3 | Wilkerson GB, Colston MA, Short NI, Neal KL, Hoewischer PE, Pixley JJ. Neuromuscular changes in female collegiate athletes resulting from a plyometric jump-training program. Journal of Athletic Training. 2004 Jan 1;39(1):17-23. | Intervention shorter than 4 weeks or sessions longer that 35 minutes |
| 4 | DiStefano LJ, Blackburn JT, Marshall SW, Guskiewicz KM, Garrett WE, Padua DA. Effects of an age-specific anterior cruciate ligament injury prevention program on lower extremity biomechanics in children. The American journal of sports medicine. 2011 May 1;39(5):949-57. | Age of participant less than 14 years old |
| 5 | Parsons JL, Alexander MJ. Modifying spike jump landing biomechanics in female adolescent volleyball athletes using video and verbal feedback. The Journal of Strength & Conditioning Research. 2012 Apr 1;26(4):1076-84. | Age of participant less than 14 years old |
| 6 | Myers CA, Hawkins D. Alterations to movement mechanics can greatly reduce anterior cruciate ligament loading without reducing performance. Journal of biomechanics. 2010 Oct 19;43(14):2657-64. | No intervention or intervention shorter than 8 sessions; |
| 7 | Louw Q, Grimmer K, Vaughan CL. Biomechanical outcomes of a knee neuromuscular exercise programme among adolescent basketball players: a pilot study. Physical Therapy in Sport. 2006 May 31;7(2):65-73. | Intervention shorter than 4 weeks or sessions longer that 35 minutes |
| 8 | Greska EK, Nelson Cortes D, Van Lunen BL, Oñate JA. A feedback inclusive neuromuscular training program alters frontal plane kinematics. Journal of Strength and Conditioning Research. 2012 Jun;26(6):1609. | Intervention shorter than 4 weeks or sessions longer that 35 minutes |
| 9 | Noyes FR, Barber-Westin SD, Smith ST, Campbell T. A training program to improve neuromuscular and performance indices in female high school soccer players. The Journal of Strength & Conditioning Research. 2013 Feb 1;27(2):340-51. | Intervention shorter than 4 weeks or sessions longer that 35 minutes |
| 10 | Noyes FR, Barber-Westin SD, Smith ST, Campbell T. A training program to improve neuromuscular indices in female high school volleyball players. The Journal of Strength & Conditioning Research. 2011 Aug 1;25(8):2151-60. | Intervention shorter than 4 weeks or sessions longer that 35 minutes |
| 11 | Noyes FR, Barber-Westin SD, Smith ST, Campbell T, Garrison TT. A training program to improve neuromuscular and performance indices in female high school basketball players. The Journal of Strength & Conditioning Research. 2012 Mar 1;26(3):709-19. | Intervention shorter than 4 weeks or sessions longer that 35 minutes |
| 12 | Grandstrand SL, Pfeiffer RP, Sabick MB, DeBeliso M, Shea KG. The effects of a commercially available warm-up program on landing mechanics in female youth soccer players. The Journal of Strength & Conditioning Research. 2006 May 1;20(2):331-5. | Age of participant less than 14 years old |
| 13 | DiStefano LJ, Padua DA, DiStefano MJ, Marshall SW. Influence of age, sex, technique, and exercise program on movement patterns after an anterior cruciate ligament injury prevention program in youth soccer players. The American Journal of Sports Medicine. 2009 Mar 1;37(3):495-505. | Age of participant less than 14 years old |
| 14 | Barber-Westin SD, Smith ST, Campbell T, Noyes FR. The drop-jump video screening test: retention of improvement in neuromuscular control in female volleyball players. The Journal of Strength & Conditioning Research. 2010 Nov 1;24(11):3055-62. | Intervention shorter than 4 weeks or sessions longer that 35 minutes |

| 15 | Vescovi JD, Canavan PK, Hasson S. Effects of a plyometric program on vertical landing force and jumping performance in college women. Physical Therapy in Sport. 2008 Nov 30;9(4):185-92. | Intervention shorter than 4 weeks or sessions longer than 35 minutes |
| --- | --- | --- |
| 16 | Leporace G, Praxedes J, Pereira GR, Pinto SM, Chagas D, Metsavaht L, Chame F, Batista LA. Influence of a preventive training program on lower limb kinematics and vertical jump height of male volleyball athletes. Physical Therapy in Sport. 2013 Feb 28;14(1):35-43. | Age of participant less than 14 years old |
| 17 | McCurdy KW, Langford GA, Doscher MW, Wiley LP, Mallard KG. The effects of short-term unilateral and bilateral lower-body resistance training on measures of strength and power. The Journal of Strength & Conditioning Research. 2005 Feb 1;19(1):9-15. | Non-athletes participants |
| 18 | Herman DC, Oñate JA, Weinhold PS, Guskiewicz KM, Garrett WE, Yu B, Padua DA. The effects of feedback with and without strength training on lower extremity biomechanics. The American Journal of Sports Medicine. 2009 Jul 1;37(7):1301-8. | No intervention or intervention shorter than 8 sessions |
| 19 | Kraemer R, Knobloch K. A Soccer-Specific Balance Training Program for Hamstring Muscle and Patellar and Achilles Tendon Injuries An Intervention Study in Premier League Female Soccer. The American journal of sports medicine. 2009 Jul 1;37(7):1384-93. | No risk factors were controlled but the incidence of injury |
| 20 | Verrall GM, Slavotinek JP, Barnes PG. The effect of sports specific training on reducing the incidence of hamstring injuries in professional Australian Rules football players. British journal of sports medicine. 2005 Jun 1;39(6):363-8. | No risk factors were controlled but the incidence of injury |
| 21 | Ono T, Fujii H. Comparison of different hamstrings training effect on hamstrings. Japanese Journal of Physical Fitness and Sports Medicine. 2013;62(1):87-94. | Non English publication (only tittle and abstract were available in English) |
| 22 | Andersson N. The Effects of a New ACL-Injury Prevention Device on Knee Kinematics and Hamstring and Quadriceps Co-Contraction: A Pilot Study.2013 | Risk factors non properly monitored |
| 23 | Sañudo B, Feria A, Carrasco L, de Hoyo M, Santos R, Gamboa H. Does whole body vibration training affect knee kinematics and neuromuscular control in healthy people?. Journal of sports sciences. 2012 Oct 1;30(14):1537-44. | Non-athletes participants |
| 24 | Shultz R, Silder A, Malone M, Braun HJ, Dragoo JL. Unstable Surface Improves Quadriceps: Hamstring Co-contraction for Anterior Cruciate Ligament Injury Prevention Strategies. Sports Health: A Multidisciplinary Approach. 2014 Dec 31:1941738114565088. | Risk factors non properly monitored |
| 25 | van der Horst N, Smits DW, Petersen J, Goedhart EA, Backx FJ. The Preventive Effect of the Nordic Hamstring Exercise on Hamstring Injuries in Amateur Soccer Players A Randomized Controlled Trial. The American journal of sports medicine. 2015 Mar 20:0363546515574057. | No risk factors were controlled but the incidence of injury |
| 26 | Petersen J, Thorborg K, Nielsen MB, Budtz-Jørgensen E, Hölmich P. Preventive effect of eccentric training on acute hamstring injuries in men’s soccer a cluster-randomized controlled trial. The American journal of sports medicine. 2011 Nov 1;39(11):2296-303. | No risk factors were controlled but the incidence of injury |

| 27 | Stearns KM, Powers CM. Improvements in hip muscle performance result in increased use of the hip extensors and abductors during a landing task. The American journal of sports medicine. 2014 Mar 1;42(3):602-9. | Non-athletes participants |
| --- | --- | --- |
| 28 | Nyland J, Burden R, Krupp R, Caborn DN. Whole body, long-axis rotational training improves lower extremity neuromuscular control during single leg lateral drop landing and stabilization. Clinical Biomechanics. 2011 May 31;26(4):363-70. | Non-athletes participants |
| 29 | Small K, McNaughton L, Greig M, Lovell R. The effects of multidirectional soccer-specific fatigue on markers of hamstring injury risk. Journal of Science and Medicine in Sport. 2010 Jan 31;13(1):120-5. | No intervention or intervention shorter than 8 sessions; |
| 30 | Verrall GM, Slavotinek JP, Barnes PG. The effect of sports specific training on reducing the incidence of hamstring injuries in professional Australian Rules football players. British journal of sports medicine. 2005 Jun 1;39(6):363-8. | No risk factors were controlled but the incidence of injury |
| 31 | Silvers HJ, Mandelbaum BR. ACL injury prevention in the athlete. Sport-Orthopädie-Sport-Traumatologie-Sports Orthopaedics and Traumatology. 2011 Dec 31;27(1):18-26. | No intervention or intervention shorter than 8 sessions; |
| 32 | Myer GD, Chu DA, Brent JL, Hewett TE. Trunk and hip control neuromuscular training for the prevention of knee joint injury. Clinics in sports medicine. 2008 Jul 31;27(3):425-48. | No intervention or intervention shorter than 8 sessions; |
| 33 | Myklebust G, Engebretsen L, Brækken IH, Skjølberg A, Olsen OE, Bahr R. Prevention of anterior cruciate ligament injuries in female team handball players: a prospective intervention study over three seasons. Clinical Journal of Sport Medicine. 2003 Mar 1;13(2):71-8. | No risk factors were controlled but the incidence of injury |
| 34 | Petersen W, Braun C, Bock W, Schmidt K, Weimann A, Drescher W, Eiling E, Stange R, Fuchs T, Hedderich J, Zantop T. A controlled prospective case control study of a prevention training program in female team handball players: the German experience. Archives of orthopaedic and trauma surgery. 2005 Nov 1;125(9):614-21. | No risk factors were controlled but the incidence of injury |
| 35 | Hewett TE, Stroupe AL, Nance TA, Noyes FR. Plyometric training in female athletes decreased impact forces and increased hamstring torques. The American Journal of Sports Medicine. 1996 Dec 1;24(6):765-73. | Intervention shorter than 4 weeks or sessions longer that 35 minutes |
| 36 | Mandelbaum BR, Silvers HJ, Watanabe DS, Knarr JF, Thomas SD, Griffin LY, Kirkendall DT, Garrett W. Effectiveness of a neuromuscular and proprioceptive training program in preventing anterior cruciate ligament injuries in female athletes 2-year follow-up. The American Journal of Sports Medicine. 2005 Jul 1;33(7):1003-10. | No risk factors were controlled but the incidence of injury |
| 37 | Myer GD, Brent JL, Ford KR, Hewett TE. A pilot study to determine the effect of trunk and hip focused neuromuscular training on hip and knee isokinetic strength. British journal of sports medicine. 2008 Jul 1;42(7):614-9. | Intervention shorter than 4 weeks or sessions longer than 35 minutes |
| 38 | Myer GD, Ford KR, PALUMBO OP, Hewett TE. Neuromuscular training improves performance and lower-extremity biomechanics in female athletes. The Journal of Strength & Conditioning Research. 2005 Feb 1;19(1):51-60. | Intervention shorter than 4 weeks or sessions longer than 35 minutes |
| 39 | Myer GD, Ford KR, Brent JL, Hewett TE. Differential neuromuscular training effects on ACL injury risk factors in. BMC musculoskeletal disorders. 2007 May 8;8(1):39. | Intervention shorter than 4 weeks or sessions longer than 35 minutes |

| 40 | Myer GD, Ford KR, McLean SG, Hewett TE. The effects of plyometric versus dynamic stabilization and balance training on lower extremity biomechanics. The American journal of sports medicine. 2006 Mar 1;34(3):445-55. | Intervention shorter than 4 weeks or sessions longer than 35 minutes |
| --- | --- | --- |
| 41 | Hewett TE, Lindenfeld TN, Riccobene JV, Noyes FR. The effect of neuromuscular training on the incidence of knee injury in female athletes a prospective study. The American journal of sports medicine. 1999 Nov 1;27(6):699-706. | No risk factors were controlled but the incidence of injury |
| 42 | Myer GD, Ford KR, Brent JL, Hewett TE. The effects of plyometric vs. dynamic stabilization and balance training on power, balance, and landing force in female athletes. The Journal of Strength & Conditioning Research. 2006 May 1;20(2):345-53. | Intervention shorter than 4 weeks or sessions longer that 35 minutes |
| 43 | Mendiguchia J, Martinez‐Ruiz E, Morin JB, Samozino P, Edouard P, Alcaraz PE, Esparza‐Ros F, Mendez‐Villanueva A. Effects of hamstring‐emphasized neuromuscular training on strength and sprinting mechanics in football players. Scandinavian journal of medicine & science in sports. 2015 Dec 1;25(6):e621-9. | Intervention shorter than 4 weeks or sessions longer that 35 minutes |
| 44 | Blackburn JT, Norcross MF, Cannon LN, Zinder SM. Hamstrings stiffness and landing biomechanics linked to anterior cruciate ligament loading. Journal of athletic training. 2013 Dec;48(6):764-72. | No intervention or intervention shorter than 8 sessions; |
| 45 | Chappell JD, Herman DC, Knight BS, Kirkendall DT, Garrett WE, Yu B. Effect of fatigue on knee kinetics and kinematics in stop-jump tasks. The American journal of sports medicine. 2005 Jul 1;33(7):1022-9. | No intervention or intervention shorter than 8 sessions; |
| 46 | McCurdy K, Walker J, Saxe J, Woods J. The effect of short-term resistance training on hip and knee kinematics during vertical drop jumps. The Journal of Strength & Conditioning Research. 2012 May 1;26(5):1257-64. | Non-athletes participants |
| 47 | Gabbe BJ, Branson R, Bennell KL. A pilot randomised controlled trial of eccentric exercise to prevent hamstring injuries in community-level Australian Football. Journal of science and medicine in sport. 2006 May 31;9(1):103-9. | No intervention or intervention shorter than 8 sessions; |
| 48 | Irmischer BS, Harris C, Pfeiffer RP, DeBeliso MA, Adams KJ, Shea KG. Effects of a knee ligament injury prevention exercise program on impact forces in women. The Journal of Strength & Conditioning Research. 2004 Nov 1;18(4):703-7. | Non-athletes participants |
| 49 | Söderman K, Werner S, Pietilä T, Engström B, Alfredson H. Balance board training: prevention of traumatic injuries of the lower extremities in female soccer players?. Knee surgery, sports traumatology, arthroscopy. 2000 Nov 1;8(6):356-63. | No risk factors were controlled but the incidence of injury |
| 50 | Croisier JL, Ganteaume S, Binet J, Genty M, Ferret JM. Strength imbalances and prevention of hamstring injury in professional soccer players a prospective study. The American journal of sports medicine. 2008 Aug 1;36(8):1469-75. | No risk factors were controlled but the incidence of injury |
| 51 | Karatrantou K, Gerodimos V, Dipla K, Zafeiridis A. Whole-body vibration training improves flexibility, strength profile of knee flexors, and hamstrings-to-quadriceps strength ratio in females. journal of Science and Medicine in Sport. 2013 Sep 30;16(5):477-81. | Non-athletes participants |
| 52 | Khodayari B, Dehghani Y. The investigation of mid-term effect of different intensity of PNF stretching on improve hamstring flexibility. Procedia-Social and Behavioral Sciences. 2012 Dec 31;46:5741-4. | No intervention or intervention shorter than 8 sessions; |

| 53 | Brockett CL, Morgan DL, Proske UW. Human hamstring muscles adapt to eccentric exercise by changing optimum length. Medicine and science in sports and exercise. 2001 May 1;33(5):783-90. | No intervention or intervention shorter than 8 sessions; |
| --- | --- | --- |
| 54 | Oliveira AD, Caputo F, Gonçalves M, Denadai BS. Heavy-intensity aerobic exercise affects the isokinetic torque and functional but not conventional hamstrings: quadriceps ratios. Journal of Electromyography and Kinesiology. 2009 Dec 31;19(6):1079-84. | Intervention shorter than 4 weeks or sessions longer than 35 minutes |
| 55 | van Beijsterveldt AM, van de Port IG, Krist MR, Schmikli SL, Stubbe JH, Frederiks JE, Backx FJ. Effectiveness of an injury prevention programme for adult male amateur soccer players: a cluster-randomised controlled trial. British journal of sports medicine. 2012 Aug 9:bjsports-2012. | No risk factors were controlled but the incidence of injury |
| 56 | Kamani, N. and Nikseresht A. The Effect of 8 Weeks Plyometric Training and 3 Weeks Detraining on Sprint, Agility and Leg Explosive Power in Female Runners: International journal of Biology, Pharmacy and Allied science. 2015 Jan:4(1):147-159 | Risk factors not properly monitored |
